# Psychosocial Support Interventions for Adult Critically Ill Patients During the Acute Phase of Their ICU Stay: A Scoping Review

**DOI:** 10.3390/healthcare13172182

**Published:** 2025-09-01

**Authors:** Usha Pant, Krooti Vyas, Elizabeth Papathanassoglou

**Affiliations:** Faculty of Nursing, University of Alberta, Edmonton Clinic Health Academy (ECHA), 11405-87th Ave., Edmonton, AB T6G 1C9, Canada; upant@ualberta.ca (U.P.); krooti@ualberta.ca (K.V.)

**Keywords:** critical illness, ICU, adults, psychosocial support, holistic intervention

## Abstract

**Background**: Addressing Intensive Care Unit (ICU) patients’ psychological well-being is crucial, yet psychosocial support interventions that can facilitate effective coping, ultimately decreasing stress-related physiological, mental health, and cognitive sequelae, are not currently included in clinical practice guidelines and standards. **Objective**: To identify and synthesize research evidence on psychosocial support interventions in the ICU, including types of outcomes and measures of effectiveness, and to explore research gaps and barriers to implementation. **Method**: The review was directed by a protocol based on current guidance for scoping reviews. The quality of studies was assessed using the National Institute for Health and Clinical Excellence. The review focused on articles containing evaluations of psychosocial interventions through an experimental or quasi-experimental design or pretest-posttest comparisons. Databases searched included Medline, CINAHL, PubMed, PsychInfo, and the Cochrane Library. **Results**: Ten highly heterogeneous studies were identified, encompassing diverse interventions (e.g., relaxation, psychotherapy, spirituality, and positive suggestions) and patient populations. Across the 10 studies, no intervention type was replicated, and most samples were small and quasi-experimental, limiting internal validity and preventing quantitative synthesis. Despite these limitations, the evidence reviewed supports that various psychosocial interventions, including positive suggestions (constructive, reassuring thoughts), relaxation techniques, psychotherapy (emotional, behavioral guidance), and spiritual and/or religious support can alleviate psychological sequelae, such as depression, anxiety, and Post Traumatic Stress in ICU patients. **Conclusions**: This review highlights the positive impact of psychosocial interventions on alleviating psychological distress in ICU patients. However, a critical gap exists in understanding their effects on other clinical and physiological outcomes, necessitating comprehensive research.

## 1. Background

The Intensive Care Unit (ICU) environment and its advanced treatment modalities can cause significant psychological distress for patients and their families, despite their life-saving impact [1,2]. Several invasive procedures in the ICU can elicit pain and be perceived as traumatic [3]. ICU patients may also experience acute stress and a range of negative emotions due to the unpredictability of their situation and fear of death [4]. Several studies have demonstrated high prevalence of anxiety and depression among ICU patients [5,6], along with sensory disruption and sleep deprivation [7,8]. Uncontrolled stress in the ICU is associated with immune, metabolic, and organ dysfunction, increased pain, and sleep disturbances [9,10,11,12]. These result in numerous physiological and psycho-cognitive complications and increased mortality during and after the ICU stay [13].

ICU patients may continue to experience distressing flashbacks of their time in the ICU even after discharge, resulting in a decline in their overall psychological health [14,15]. Depression, anxiety, and stress are prevalent in more than half of ICU survivors and may last for months up to several years post-ICU [16,17,18,19]. Likewise, the prevalence of mental health disorders, including mood and personality disorders, substance use, Post Traumatic Stress Disorder (PTSD), psychosis, and schizophrenia, seems to be significantly higher in post-ICU survivors than in the general population [20].

Additionally, the stress experienced during an ICU stay is a factor in the post-intensive care syndrome (PICS). PICS is a culmination of cognitive, physical, and psychological impairments that persist after critical illness [21]. It is prevalent in approximately 65% at three and six months and 55% at one year after ICU discharge [22,23]. PICS can lead to readmission to the ICU, propagating a vicious circle of deterioration of well-being [24].

Addressing the psychological well-being of ICU patients is an important aspect of patient-centered care. However, current clinical guidelines and practice standards [25] focus primarily on physical recovery, with limited emphasis on psychological support. Pharmacological interventions, such as sedatives, are commonly used to manage stress in the ICU, but they are often ineffective and carry significant side effects [26,27]. Similarly, the current post-ICU care framework includes follow-up clinics and rehabilitation programs to support ICU survivors in their recovery. However, evidence suggests that the current approaches are ineffective [28]. Hence, integrating psychological support interventions during ICU stays is vital in preventing psychological distress and improving long-term outcomes in survivors.

Providing psychosocial support can improve mental health outcomes by helping patients and their families cope more effectively. Furthermore, improving the mental health of critically ill patients may also enhance physical recovery and reduce the strain on ICU resources. Psychosocial support refers to interventions that address the emotional, psychological, and social needs of critically ill patients and their families [29]. In ICU settings, this includes strategies such as stress management, family engagement, and mental health support to reduce distress and promote coping [30].

Psychosocial support interventions are grounded in stress-and-coping theory, which highlights how emotional and social support can reduce the negative effects of critical illness by promoting effective coping [31]. In nursing, Kolcaba’s Comfort Theory supports these interventions by emphasizing the importance of relieving discomfort and promoting a sense of ease and well-being, especially in high-stress settings like the ICU [32]. Neuman’s Systems Model also offers a relevant framework, viewing psychosocial support as a way to strengthen patients’ adaptive capacity against stress and restore balance across physical, emotional, and social dimensions [33].

This study employs a scoping review methodology to systematically identify and synthesize existing evidence on psychosocial support interventions in the ICU. This approach was chosen for its ability to map the breadth and nature of research on psychosocial interventions for critically ill patients [34]. Thus, facilitating a comprehensive synthesis of current knowledge, thereby informing future research priorities and guiding evidence-based clinical practice.

### Purpose

The purpose of this study was to identify and synthesize research evidence on psychosocial support interventions in the ICU. Specific objectives included identifying (a) types of interventions studied, (b) types of outcomes assessed, (c) measures of effectiveness of interventions, (d) research gaps, and (e) identified barriers to implementation. We hope to provide a synthesis for healthcare providers seeking to implement these interventions in their practice and to motivate impactful change in the field of psychosocial health and well-being in critical care. For this work, we defined psychosocial interventions as interpersonal or informational approaches that target behavioral, cognitive, emotional, interpersonal, or social factors aiming to improve coping and well-being [35].

## 2. Methods

The review was directed by a protocol based on current guidance for scoping reviews [36] and registered with the Open Science Framework (https://doi.org/10.17605/OSF.IO/53FUE). Reporting was guided by Preferred Reporting Items for Systematic Reviews and Meta-Analyses extension for Scoping Reviews (PRISMA-ScR) [37]. The review was guided by the following question, based on the Population/Concept/Context format [36]: “What psychosocial interventions have been explored involving adults during the acute phase of ICU hospitalization, and what is the evidence of their effectiveness and feasibility within the context of psychological distress in the ICU?”

### 2.1. Eligibility Criteria

Inclusion criteria: We included studies involving:

1. Primary studies of quantitative, qualitative, or mixed methods, reporting on any type of psychosocial intervention in ICU patients.
2. Studies involving interventions for adult intensive or critical care patients while still hospitalized in a critical/intensive care unit, regardless of their primary diagnoses and type of unit.
3. Peer-reviewed studies published since 2009 were included to capture evidence following the introduction of key ICU guidelines on pain, agitation, and delirium, which significantly changed clinical practice. This cut-off ensures the review reflects current standards of care.

Exclusion criteria: We excluded studies involving:

1. Case studies, literature reviews, opinion pieces, editorials, research protocols
2. Intervention(s) for family members only, not involving ICU patients
3. Patients under 18 years of age, such as neonates, infants, children, and paediatric patients.
4. Articles in any language other than English.

### 2.2. Information Sources and Search Strategy

Databases searched included Medline, CINAHL, PubMed, PsychInfo, and the Cochrane Library. Moreover, hand searches of reference lists and forward citation searches were conducted to ensure all key articles are included. Grey literature and trial registries were not included in this review to ensure consistency and quality of evidence from peer-reviewed, published studies. The review focused on articles containing evaluations of psychosocial interventions, either through an experimental or quasi-experimental design or pretest-posttest comparisons. We employed a combination of natural language vocabulary and controlled terms derived from two main concepts: (a) ICU and (b) Psychosocial interventions. No sample size or outcome measures limitations were set. Studies were retrieved through online database searches, constructed through consultation with a library information specialist. References of identified studies were also checked for relevancy. For Ovid/MEDLINE, the following search strategy was used: ((“Intensive care” OR “critical care” OR “critical ill*” OR ICU) AND (patient*) AND (Psychological* OR mental* OR emotion*) adj3 (stress OR distress OR pressure OR strain OR tension OR anxiet* OR anxiousness OR worry OR worrie* OR nervous*) AND ((Psychosocial OR spiritual* OR religious OR religion) adj3 (intervention* OR support*) OR counsel* OR “positive suggestion*” OR “positive messag*” OR mindfulness OR meditat*)). The complete search strategy, including controlled vocabulary terms (e.g., MeSH) and Boolean operators for Ovid/MEDLINE, is provided in Appendix A.

### 2.3. Screening and Data Extraction

The studies retrieved from database searches were exported into Covidence (https://www.covidence.org/) for data management, removal of duplicates, and independent screening by reviewers. Two team members independently screened titles and abstracts of search outcomes for eligibility, and conflicts were resolved with a third reviewer. Articles that were deemed relevant for the topic under study were passed on to the full-text screening. Two reviewers screened independently; disagreements were resolved by a third reviewer. No kappa statistic was calculated. A data charting tool was iteratively developed by the research team and pilot tested on the first five articles to ensure all required data was captured. We extracted data on participant demographics such as age, sex, and race; ICU type; outcome variables; assessment tools intervention details such as frequency and duration and statistical/qualitative findings. Data was independently extracted by two reviewers using a data charting tool to ensure consistency and completeness. Any discrepancies or conflicts were resolved through a third reviewer. Following data extraction, the review team met to conduct a structured analysis, categorizing interventions, outcomes, and study characteristics. Patterns, gaps, and trends within the evidence were then synthesized to provide a comprehensive overview of psychosocial support interventions in the ICU.

### 2.4. Quality Assessment

The quality of studies was assessed using the National Institute for Health and Clinical Excellence (NICE) Checklist (2012), which contains five domains and evaluates aspects of internal and external validity [38]. The process was iterative and continued until consensus among 3 raters was achieved. Quality appraisal was used to inform synthesis and interpretation of findings. No studies were excluded based on the results of quality appraisal.

## 3. Results

**Figure 1:**
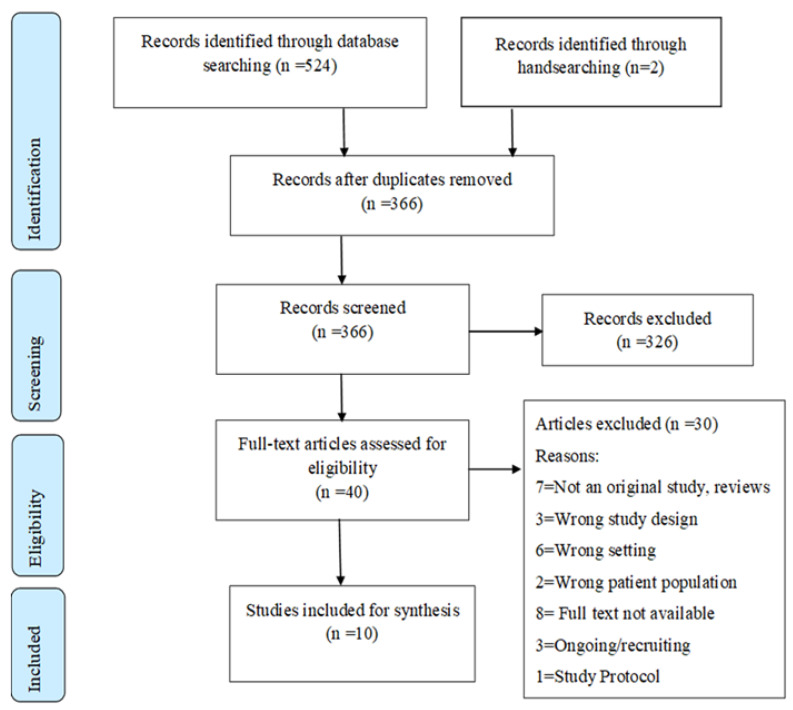
PRISMA Chart for Scoping Review.

Out of the initial 524 studies identified (MEDLINE = 232, CINAHL = 159, PubMed = 47, PsychInfo = 38, Cochrane review = 48), 160 duplicates were removed. Including two articles identified through manual hand-searching, 366 studies underwent title and abstract screening, with 40 entering the full text review stage. Ten studies were ultimately selected for data extraction (Figure 1).

### 3.1. Study Characteristics

Half of the studies (n = 5) were conducted in the United States [39,40,41,42,43], while the rest took place in Iran, (n = 1) [44], Hungary (n = 1) [45], Germany (n = 1) [46], China (n = 1) [47], and the United Kingdom (n = 1) [48] (Table 1). All studies included some sort of support intervention, including positive suggestions [41,42,45], relaxation techniques [39,43], spiritual and/or religious support [40,44], and psychotherapy-based [46,47,48]. In general, the samples were primarily white and Christian.

Of the identified ten studies, the majority adopted a quasi-experimental pretest-posttest design (n = 6) [40,41,42,43,44,47], three were randomized controlled trials (RCTs) [39,45,46], and one was a mixed methods study [48]. Of the studies with quasi-experimental design, only two studies used control groups [42,44]. The number of participants ranged from 26 to 76, and samples were mostly male (Table 1). The outcomes addressed included measures of effectiveness of interventions with regard to improving psychological well-being, including anxiety, stress, pain, social support, depression, anxiety, delirium, length of ICU stay, length of mechanical ventilation, and feasibility and acceptability measures.

Of the three RCTs, Heilmann et al. (2016) recruited 253 Coronary Artery Bypass Grafting (CABG) patients, mostly male [46]. Bannon et al. (2020) recruited 16 neuro-ICU patients with similar male/female distribution [39]. Szilágyi et al., 2014 recruited 26 ICU patients requiring mechanical ventilation, mostly male [45]. RCTs focused on outcomes such as depression, anxiety, post-traumatic stress (PTS), length of ICU stay, length of mechanical ventilation, and feasibility/acceptability. The only mixed-methods study did not use a control group (Wade et al., 2018) and addressed the feasibility and acceptability of a stress management intervention in ICU patients [48]. The study recruited 127 patients and did not report on the age and sex/gender of the participants.

### 3.2. Quality Appraisal of Included Studies

The results of the quality appraisal of the included studies are demonstrated in Table 2. The studies exhibited a mixed level of quality. As expected, all quasi-experimental studies displayed a high risk of bias in terms of randomization, allocation concealment, outcome measures, and analysis. Furthermore, the absence of sufficient descriptions regarding the study approaches posed challenges in determining the overall quality of the included studies. However, no studies were excluded from the synthesis on the basis of methodological quality, ensuring a comprehensive and inclusive evidence base.

The majority of the articles did not provide information on the allocation method employed for assigning participants to either the intervention or control group, so selection bias cannot be excluded [40,41,42,43,44,47]. Only four studies effectively minimized internal threats to validity through randomization to group allocation [39,45,46,48]. Among these, only three RCT studies implemented measures to maintain allocation concealment [39,45,46]. All included studies furnished sufficient details regarding the intervention utilized and the characteristics of both the comparison and intervention groups. Additionally, all studies employed comparable follow-up durations for both groups. However, due to the inherent nature of the interventions, blinding of participants and/or personnel was not feasible, resulting in a high risk of performance bias across all studies.

All studies employed reliable outcome measures and evaluated important outcomes relevant to the study. Moreover, all studies utilized appropriate analysis methods and provided sufficient data for effect size calculation, with the exception of one study [48]. Only one study had sufficient power and reported effect size data [46]. However, the majority of studies (n = 7) did not report estimates of effect size [39,40,41,43,47,48]. Additionally, two studies had inadequate sample sizes to detect any intervention effects [42,44]. In three studies (not all participants were included in the analysis, and it remained unclear whether an intention-to-treat analysis was applied [39,42,46]. Upon analysis, only one study demonstrated poor internal validity [44], while another study exhibited limitations in terms of generalizability [41].

### 3.3. Types of Interventions

The identified interventions were based on positive suggestions [41,42,45], psychotherapy techniques [46,47,48] relaxation techniques [39,43], spiritual and/or religious support [40,44] (Table 3). Each intervention differs in its approach and techniques, and no interventions have been replicated in this body of evidence. All interventions were delivered during patients’ ICU stay.

Spiritual-religious-based interventions, facilitated by chaplains or researchers, emphasize spiritual care, worship, and assessing spiritual needs, typically lasting from 25 min to 90 min. The *Picture-guided spiritual care* intervention (25 min; delivered once) involved chaplain-led sessions using communication cards to assess spiritual affiliation, emotions, and needs in conscious ICU patients requiring mechanical ventilation [40]. Similarly, another spiritual/religious-based intervention delivered by researchers (60–90 min for 3 days) included caring presence, instilling hope, encouraging generosity, strengthening personal relationships, and providing opportunities for worship and prayer in coronary care unit patients [44].

Positive suggestion-based interventions, administered by psychotherapists, nurses, study intensivists, ICU doulas, or even delivered via pre-recorded messages through MP3 players, focused on building confidence and rapport, exploring fears, and normalizing ICU experiences, with sessions ranging from 5 to 30 min. Similarly, the *Psychological Support Based on Positive Suggestions* (PSBPS) intervention (5–12 min, once daily throughout the patient’s ICU stay) involved physicians developing rapport and therapeutic touch with patients, addressing fears, and normalizing the ICU experience, delivered by study intensivists [41,42]. It consisted of a debriefing with information on what to expect after transferring to a recovery ward and an opportunity to explore fears. Szilágyi et al. (2014) used prerecorded text focused on promoting recovery and healing delivered via MP3 players and headphones to ventilated ICU patients (30-min sessions, once daily throughout their ICU stay) [45].

Psychotherapy-based interventions often overlapped with other interventions, as they included relaxation and/or information-delivery sessions. A psychotherapeutic intervention (15–30 min, 3 days/week) provided by a psychotherapist consisted of listening, positive attention, supportive psychotherapy, empathy, muscle and breath relaxation, and cognitive behavioral therapy [47]. The *Provision Of Psychological Support to People in Intensive Care* (POPPI) consisted of three stress support sessions and a relaxation and recovery program provided by trained staff [48]. Sessions started within 48 h of the Intensive Care Psychological Assessment Tool (IPAT) screening, and a total of three sessions were delivered. A psychotherapeutic intervention (30 min, once before CABG) by Heilmann et al. (2016) comprised open dialogue, relaxation exercises, and emotional support, provided by trained nurses one day prior to CABG [46]. This intervention also included information regarding surgery, postoperative care, pain management, ICU stay, and emotional support.

Relaxation-based interventions, led by clinicians, study staff, or trained nurses, concentrate on stress management, breath control, coping skills, and psychosocial impact assessment, with sessions lasting from 20 to 30 min and occurring multiple times per week. The *Recovering Together* (20–30 min) included elements of cognitive behavioral therapy, dialectical behavioral therapy, and trauma-informed care [39]. Tangible facets of this intervention focused on deep breathing, meditation, self-care, stress management, and appropriate communication. A similar guided virtual VR-based meditative intervention, *RelaxVR* (5–20 min, 7 days), provided by study staff, consisted of calm immersive scenes with voice-guided meditation that promoted breath control and relaxation [43].

#### 3.3.1. Psychological Well-Being Outcomes

We narratively synthesized the results, and we calculated effect sizes where pertinent data were available (Table 4).

Bannon et al. (2020) conducted a dyadic intervention, *Recovering Together*, for stroke patients and their caregivers who were at risk for chronic emotional distress such as depression, anxiety, and PTS (N = 16; Intervention (I) = 7 and Control (C) = 9) [39]. Outcomes were assessed post intervention during ICU stay and 3 months post discharge from ICU. The control group received minimally enhanced usual care, including an informational pamphlet on the stroke experience and post-stroke recovery for patients and their informal caregivers. *Recovering Together* showed evidence of being a feasible and acceptable intervention in reducing risk for chronic anxiety and post-traumatic stress in both stroke patients and caregivers during ICU. Within-group effect sizes demonstrated improvement in all emotional distress outcomes (decrease in symptoms of depression, anxiety, and post-traumatic stress) between baseline and post-test with medium to very large effect sizes in the ICU patients (anxiety, Cohen’s d (*d*) = −1.25, post-traumatic stress, *d* = −0.83, and depression, *d* = −0.42). Additionally, when compared with the control group, from baseline to post-intervention (during ICU) to 3 months, a general improvement in emotional distress for dyads in the intervention group and a general deterioration in symptoms for those in the control group was noted. However, the very small sample size restricts confidence in any between-group comparisons. In another RCT, Heilmann et al. (2016) developed a 30 min intervention rooted in psychotherapy to provide emotional support for CABG surgery patients in the ICU (N = 253; I = 139 and C = 114) [46]. Significant decreases were only noted for State-Trait Operation Anxiety within the intervention group when compared with the control group. We calculated effect sizes of the intervention, based on reported data. We noted medium to very small effect sizes immediately after intervention (cognitive anxiety, *d* = −0.39, affective anxiety, *d* = −0.125 and Visual Analog Scale (VAS), *d* = −0.20). On postoperative day 5, the effect size for VAS was small (*d* = −0.28) and very small for affective anxiety (*d* = −0.125) and cognitive anxiety (*d* = −0.17). This nurse-delivered intervention is feasible for CABG patients; although its impact on anxiety was modest, it offers a low-intensity model warranting further evaluation in larger trials.

Wade et al. (2018), through a patient interview, demonstrated that psychological support intervention by trained nurses can develop appropriate coping strategies in ICU patients (10 of 15 patients) [48]. Among 25 participants, stress measured through a stress thermometer illustrated a reduction of three points (IQR = 1–5 points) in just 3 sessions, but the reported data are not appropriate for an effect size calculation.

Similarly, Ong et al. (2020) explored the effects of guided virtual reality-based meditative intervention on pain, sleep quality, affect, and delirium in surgical and trauma ICU patients [43]. The intervention showed statistically significant improvement in anxiety and depression symptoms after two sessions. Moreover, results of the patient questionnaires revealed that the intervention was comfortable, enjoyable, and relaxing. Yang et al. (2020) investigated the effect of an in-person relaxation and support approach on sleep, depression, anxiety, and social support among patients with COVID-19 in an isolated ICU [47]. Pre-post-intervention comparisons demonstrated the potential of the intervention to significantly reduce depression and anxiety. However, the reported mean scores for PSQI (11.20 vs. 49.16), PHQ-9 (8.80 vs. 52.15), and GAD-7 (10.69 vs. 63.86) exceeded the possible ranges of these instruments (PSQI: 0–21; PHQ-9: 0–27; GAD-7: 0–21). These values are presented as reported in the original publication and should be interpreted with caution.

Elham et al. (2015) used spiritual- and religious-based interventions in ICU patients and showed the potential of spiritual-based interventions to significantly reduce anxiety during the ICU stay [44]. The study results indicated that the spiritual/religious intervention could enhance spiritual well-being and reduce state anxiety and trait anxiety in the elderly admitted to the Coronary Care Unit (Table 1), but the reported data were not sufficient for an effect size calculation. Similarly, Berning et al. (2016) demonstrated that spiritual-based interventions can significantly reduce anxiety and stress during the ICU stay [40]. Patients reported an immediate reduction in anxiety post-intervention, and the outcome persisted even after the ICU discharge, as testified by follow-up with the ICU survivors.

Tan et al. (2020) demonstrated that positive suggestions can reduce psychological distress in mechanically intubated patients, but the data were not adequate to calculate effect size [42]. Similarly, Karnatovskaia et al. (2021) determined that after extensive training, doulas can effectively provide PSBPS to the ICU patients [41]. Though quantitative data were not collected, patient interviews and feedback strongly indicated that PSBPS offered profoundly reassuring support, thereby reducing ICU patients’ distress.

#### 3.3.2. Other Clinical Outcomes

Only three studies focused on clinical outcomes in ICU patients. Szilágyi et al. (2014) found significant reductions in both length of ICU stay (LOS) and length of mechanical ventilation in ICU patients [45]. Effect sizes, calculated from study data, were very large for both LOS (*d* = 1.26) and length of mechanical ventilation (*d* = 1.46) compared to controls. From the available data, we calculated the effect size comparing the suggestion group versus the music group and found a medium to large effect size (LOS: *d* = 0.58; length of mechanical ventilation: *d* = 0.69). While these results are promising, the small sample size and marginal *p*-values indicate that the findings should be interpreted with caution.

Yang et al. (2020) demonstrated the potential of in-person relaxation and support approaches to significantly improve sleep among patients with COVID-19 in an isolated ICU [47]. However, Ong et al. (2020) reported that guided virtual reality-based meditative intervention showed no significant improvement in pain, sleep, and delirium scores in surgical and trauma ICU patients [43]. The reported data were not sufficient for effect size calculations.

#### 3.3.3. Feasibility and Acceptability

Tan et al. (2020) and Karnatovskaia et al. (2021) explored the effectiveness of PSBPS intervention to reduce distress in the ICU patients [41,42]. Both identified that various stakeholders, including patients, families, and providers, regarded the intervention as useful and effective. Tan et al. (2020) demonstrated the efficacy and feasibility of PSBPS to reduce psychological distress in mechanically intubated patients [42]. Karnatovskaia et al. (2021) determined that after extensive training, doulas can effectively provide PSBPS to the ICU patients [41].

Berning et al. (2016) confirmed that the spiritual-based interventions are feasible for ICU patients [40]. Furthermore, the patient interview confirmed that the intervention was well accepted and assisted patients in dealing with the unpredictability of their illness.

Wade et al. (2018) provided psychological support to ICU patients by delivering three stress support sessions and a relaxation and recovery program [48]. The POPPI intervention was administered by trained nurses. Additionally, follow-up interviews revealed that the stress support sessions were welcomed by intervention administrators, patients, and staff. Results from the patient satisfaction questionnaire determined that patients were highly satisfied and appreciated the intervention. Furthermore, nurses’ interviews testified that the training had raised awareness, changed staff thinking, and led to better unit environments.

## 4. Discussion

We attempted a synthesis of a very heterogenous body of research exploring the effects of psychosocial interventions during ICU hospitalization. Heterogeneity stemmed from the designs, types, and delivery of interventions and participant populations and characteristics. The majority of included studies used quasi-experimental design, of which four used no control group and two were feasibility studies, and all had small sample sizes, resulting in a high risk of bias. Overall, our analysis unveiled a scarcity of studies investigating psychosocial interventions in ICU patients, comprising only 3 randomized controlled trials, of which only one was adequately powered to explore effectiveness. Despite these shortcomings, the evidence reviewed supports that various psychological interventions, including positive suggestions, relaxation techniques, psychotherapy, and spiritual and/or religious support, can alleviate psychological sequelae, such as depression, anxiety, and PTS in ICU patients. There is limited evidence to support effects on sleep in ICU patients, as only two studies included this outcome, and only one study suggested an effect on ICU LOS and length of mechanical ventilation. Although some identified interventions may not have received attention in evidence syntheses, the evidence reviewed here highlights the need to further explore these modalities to improve holistic care in the ICU.

Although few studies have assessed the feasibility and acceptability of interventions, evidence to date shows that psychological support interventions are feasible, well accepted, and associated with higher patient satisfaction. Additionally, a gender disparity in the samples was noted, with a predominant male participation. Moreover, the majority of participants in the identified studies were Caucasian and Christians, which raises the need to further explore the effectiveness and transferability of these results in diverse samples. A wide range of interventions was explored with great diversity in the way of delivery, including who delivered the interventions and their duration, timing, and frequency. There was no replication of interventions across studies, which may limit the validity of individual interventions.

The majority of identified studies lacked clear reporting on intervention duration and frequency, limiting the interpretability of their findings and making replication challenging. Future research should adopt standardized reporting frameworks, such as theTemplate for Intervention Description and Replication (TIDieR) [49], to provide precise details on intervention duration, frequency, and total sessions, thereby enhancing reproducibility and facilitating clinical translation. Additionally, there was considerable variability among the included studies with regard to outcome assessment tools used. We strongly recommend that future research employ standardized and validated tools to improve consistency, facilitate comparability across studies, and enable more robust evidence synthesis.

Despite the methodological limitations noted, these findings are important, as stress may prolong recovery times and increase the risk of complications in critically ill patients [9,10,12]. The only prior review in this area, a 2010 critical review by Papathanassoglou, examined studies published before 2009 and highlighted the potential of interventions such as psychological support, communication strategies, and sensory stimulation for ICU patients [50]. Since 2009, the introduction of key ICU guidelines on pain, agitation, and delirium has significantly shaped clinical practice, promoting more patient-centered care and increasing the use of non-pharmacologic interventions. Acknowledging the paramount effect of stress, the Society of Critical Care Medicine (SCCM) also advocates for exploring psychosocial interventions for alleviating ICU-related outcomes [51]. Given these shifts and the expanding evidence base, this scoping review is well warranted to synthesize recent research, address implementation challenges, and inform effective psychosocial support strategies in modern ICUs.

Strengthening coping mechanisms has the potential to alleviate the intensity of the stress response, interrupting the cascade of physiological and psychological responses that stem from the psychological burden of stress. Positive suggestions, delivered during sedation or mechanical ventilation, can help reduce threat appraisal and catastrophic thinking, thereby lowering anxiety and promoting a sense of safety. Findings of a meta-analysis of 26 RCTs suggest that positive suggestion techniques might be useful to alleviate anxiety in postoperative patients [52]. Similarly, supportive psychotherapy may target maladaptive coping patterns, reducing avoidance and hyperarousal that contribute to post-traumatic stress symptoms. A systematic review and meta-analysis demonstrated that positive psychological responses, such as optimism and positive affect, are significantly associated with the health outcomes and mortality in CABG patients [53]. Furthermore, relaxation-based interventions, including guided imagery, can modulate autonomic arousal, leading to improvements in pain perception, sleep quality, and overall psychological well-being. Through this theoretical scaffolding, it becomes evident why such interventions may influence psychological outcomes such as anxiety, PTSD, or sleep while having limited direct impact on physiological endpoints such as delirium, ventilator days, or ICU length of stay.

Spirituality can help to transcend suffering, pain, distress, and despair. Our review demonstrated that providing opportunities for worship and prayer can help spiritual well-being of ICU patients and reduce anxiety. It has been proposed that spiritual care practices help facilitate meaning-making out of the despair of illness [54,55,56]. Additionally, surveys [57] and reviews [58,59] in ICU patients, including post-cardiac arrest patients [60], have demonstrated that spirituality/religiosity plays a significant role in coping with crises and nurturing optimism. Despite the importance of addressing spiritual needs, spiritual care is not commonly incorporated into daily critical care practice [59], and it may often be considered part of end-of-life care [59,60,61,62].

Our review also indicates the dearth of evidence on psychosocial support interventions aimed at managing clinical outcomes such as delirium, pain, sleep, and length of ICU stays in the ICU patients [43,47]. While the evidence supported the effectiveness of in-person relaxation and support approaches in enhancing sleep among isolated COVID-19 patients, there remains a scarcity of data concerning other crucial outcomes such as delirium, pain, and the duration of ICU stay. However, studies in other acute care populations have yielded promising results for patient survival both during hospitalization and after discharge through psychosocial support interventions [63,64]. Additionally, psychosocial interventions were found effective in enhancing overall quality of life when compared with conventional therapies in traumatic brain injury patients [65]. Moreover, a recent systematic review further supports the positive impact of psychosocial interventions, revealing beneficial effects on the quality of life, emotional distress, and existential distress in terminally ill patients [66].

Positive suggestion-based interventions seem to be an emerging approach. In our review, we identified one RCT and two feasibility studies on positive suggestion-based interventions. These studies support the effectiveness of positive suggestion-based interventions in improving clinical outcomes, including decreasing LOS, speeding weaning from mechanical ventilation, and improving chances of survival [45] and decreasing psychological distress [42]. Existing evidence also supports that positive suggestions can diminish pain, decrease medication intake, improve chances of survival, and influence physiological factors, such as blood pressure during surgery [52,67,68,69,70].

Our review also highlights the need for a shift in current ICU practices to embrace holistic care that focuses on both the psychological and physical aspects of critical illness. This gap is, in part, a result of the absence of clinical practice guidelines addressing stress assessment and management [71]. The Canadian Standards of ICU Nursing (2017) currently places a higher emphasis on physiological outcomes and comfort, often sidelining crucial psychological aspects of patient care [72]. In contrast, both the American Association of Colleges of Nursing (AACN, 2019) [73] and UK competency standards [74] appear to be more inclusive. The AACN standards, rooted in the synergy model, advocate for a holistic approach, placing significant emphasis on the psychological well-being of patients [73]. AACN guidelines recommend the provision of spiritual care to ICU patients; however, this remains limited to end-of-life needs [75]. Similarly, the UK competency standards adopt a comprehensive view that recognizes the intertwined nature of both physiological and psychological dimensions [74]. In alignment with AACN and UK standards, the SCCM’s Pain, Agitation/Sedation, Delirium, Immobility, and Sleep guideline (PADIS) [76] recommends the ABCDEF care bundle, such as sedation minimization, delirium prevention, and family engagement. However, the focus of PADIS is predominantly physiologic, with limited attention to the comprehensive psychological needs of critically ill patients. While bundled physiological ICU care, such as the ABCDEF care bundle, is increasingly standardized, psychosocial interventions remain experimental and are not yet established as standard practice. The European Federation of Critical Care Nurses specifies “patient comfort and psychological care” as an important competency but provides no elaboration of the specific approaches [77]. There is a pressing need for these guidelines to align with a patient-centric approach to intensive care, emphasizing the profound impact of addressing psychological well-being on overall patient outcomes.

## 5. Research Implications

Psychosocial support interventions are clinically relevant in ICU settings, addressing the emotional, cognitive, and social needs of patients and families. However, their integration into routine care is limited by several barriers, including insufficient mental health staffing, competing clinical priorities, and a lack of standardized protocols [78,79]. Organizational culture, time constraints, and inconsistent leadership support further hinder implementation. To facilitate uptake, psychosocial care must be embedded within ICU workflows through defined roles, interdisciplinary training, and structural support. Engaging interdisciplinary team members, such as ICU doulas or allied health staff, can make interventions more practical and sustainable by reducing the workload on bedside nurses. Additional strategies, including brief “micro-sessions” (≤20 min), structured scripts, fidelity checks, and alignment with ICU rounds, can help ensure consistent delivery. Future research should not only evaluate intervention outcomes but also focus on validating standardized interventions, assessing implementation across diverse healthcare contexts, and incorporating both clinical and economic outcomes. Specifically, multicenter trials should pair validated psychological measures with delirium incidence and duration, as well as sedative exposure, prespecify clinically meaningful differences, and adopt fidelity monitoring for intervention delivery. Furthermore, studies should incorporate equity considerations—including sex/gender, socioeconomic position, culture and language, and geography—to develop scalable, culturally sensitive, and representative ICU interventions.

## 6. Limitations

The conclusions of this scoping review are limited by the heterogeneity and quality of the individual studies, as well as by small sample size. The predominantly White and Christian composition of study samples further constrains the generalizability of the findings. In addition, we only focused on English publications and those published after 2009, introducing the potential to have excluded studies that might have otherwise met these inclusion criteria. Our focus on the last 15 years was meant to capture the most recent evidence and avoid pooling current studies with older ones, which may represent very different contexts of care, because the context of care was different prior to publication of clinical practice guidelines regarding pain, agitation, and delirium in the ICU.

## 7. Conclusions

The review highlights the feasibility and positive outcomes associated with psychosocial interventions delivered during ICU stays but also notable methodological limitations in the identified studies, with a dearth of full RCTs. Psychological support approaches include modalities such as positive suggestions, relaxation, and spiritual or religious support.

We noted a critical gap in understanding the impact of psychosocial interventions on clinical outcomes such as delirium, pain, length of mechanical ventilation, and ICU LOS. The review raises concerns about the lack of diversity and representation in research participants, particularly regarding gender and cultural backgrounds. This points to the importance of ensuring that interventions are applicable and effective across diverse populations to promote inclusivity in critical care practices.

The results emphasize the necessity of a holistic shift in ICU practices, advocating for the importance of recognizing and integrating the interconnected nature of physical and psychological well-being. The shift could involve integrating psychosocial interventions into routine patient care to deliver patient-centered care in the ICU setting. Therefore, there is a need for more comprehensive research to generate high-quality evidence on both clinical and economic outcomes. Future multicenter trials should pair validated psychological measures with delirium incidence/duration and sedative dose, prespecify clinically meaningful differences, and adopt fidelity monitoring for intervention delivery.

## Figures and Tables

**Table 1:** Description and effectiveness of psychosocial interventions.

Author (Year, Country)	Study Design	Aim of the Study	Sample Size and Patient Characteristics	Intervention Details (Agent, Recipient, Content, Dose/Intensity, Delivery Mode, Setting, Training)	Outcome Variables and Measurement Tools	Analysis Tools	Main Findings	Study Conclusions
Bannon et al. (2020, United States) [39]	Randomized Controlled Trial (RCT)	“Measure feasibility and acceptability markers (primary outcomes), including ability to recruit and retain dyads, acceptability of randomization, credibility, and satisfaction.”	*# of participants:* 16 dyads from a neuro-ICU. 7 allocated to receive RT intervention; 9 allocated to receive MEUC. *Mean age:* Patients- 53.9 (±17.4) years. Caregivers- 48.88 (±10.62) years. *Gender:* Patients- 9 (56.3%) female. Caregivers- 12 (75.0%) female. *Race:* Patients- 14 (87.5%) White; 2 (12.5%) Asian. Caregivers- 13 (81.3%) White; 2 (12.5%) Asian; 1 (6.3%) identified as more than one race.	**Agent**: Unspecified clinician (discipline not specified). **Recipient**: Stroke patients and their caregivers (dyads) with at least one person (patient or caregiver) at risk of chronic emotional distress **Content**: Dyadic intervention incorporated elements of cognitive behavioral therapy (CBT), dialectical behavioral therapy (DBT), and trauma-informed care. Sessions focused on: Deep breathing Meditation Self-care strategies Stress management techniques Effective communication skills Coping with change Developing and maintaining new habits **Dose/Intensity**: Length: 20–30 min per session Frequency: 6 sessions Duration: Baseline (pre-test) to 6 weeks (post-test) **Delivery Mode**: Not explicitly stated; likely face-to-face (unspecified). **Setting**: Neuro-ICU **Training**: No training requirements for the interventionist described.	*Outcome variables:* Depression, anxiety, and post-traumatic stress (PTS) *Measurement tools:* Hospital Depression and Anxiety Scale (HADS-D, HADS-A), PTSD Checklist-Civilian Version (PCL-C), General Self-Efficacy Scale (GSE), Measure of Current Status Part A (MOCS-A), Cognitive and Affective Mindfulness Scale-Revised (CAMS), and Intimate Bond Measure (IBM). Others included Credibility and Expectancy Questionnaire (CEQS) and the Client Satisfaction Questionnaire (CSQ-3). CSQ-3 was measured at post-test.	Cohen’s *d* for effect size	Participants receiving RT showed a decrease in symptoms of depression, anxiety, and PTS in patients (*d* = −0.41, −1.25, −0.83, respectively) and caregivers (*d* = −0.63, −0.81, −0.98, respectively) from baseline to post-test. Participants who received only MEUC showed clinically significant increases in symptoms of depression, anxiety, and PTS in patients (*d* = 0.98, 0.44, 0.68, respectively) and in caregivers (*d* = 0.71, 0.48, 0.46, respectively). Resiliency variables (e.g., self-efficacy, mindfulness, perceived coping) showed small to large effect sizes (*d* = 0.07–0.85) in those who received the intervention but not in the control group from baseline to post-test.	There is “promising evidence” that the RT intervention is a feasible and acceptable program for emotional distress in stroke patients and their caregivers when compared with MEUC.
Berning et al. (2016, United States) [40]	Quasi-experimental	“To determine the feasibility and measure the effects of chaplain-led picture-guided spiritual care for mechanically ventilated adults in the ICU.”	*# of participants:* 50 patients in total. No control group was identified in this study. *Mean age:* 59 (±16) years *Gender:* 28 (56%) male *Race:* 25 (50%) White, 12 (24%) Hispanic, 9 (18%) Black, 4 (8%) Southeast Asian	**Agent**: Chaplain. **Recipient**: “Mechanically ventilated adults in medical or surgical ICUs without delirium or dementia” **Content**: Picture-guided spiritual care intervention, in English or Spanish, using illustrated communication cards to: Assess spiritual affiliation Explore emotions and needs (including spiritual pain) Identify desired religious, spiritual, or nonspiritual support the chaplain could provide Deliver tailored spiritual intervention accordingly **Dose/Intensity**: Length: Total intervention less than 25 min: 8.5 min (median; range 5–13) to complete communication card sections 18 min (median; range 11–25) for chaplain to use the card and provide specific intervention Frequency: Not reported Duration: Not reported **Delivery Mode**: Face-to-face, chaplain-led session using illustrated communication cards. **Setting**: Medical and surgical ICU **Training**: Not reported	*Outcome variables:* Anxiety, stress *Measurement tools:* 100-mm Visual Analog Scales (VAS)	Wilcoxon signed-rank test	*Pre-test* vs. *post-test:* Mean VAS score = 64 (±29) vs. 44 (±28) (absolute reduction, *p* = 0.002; relative reduction, *p* = 0.001). Mean score changed by 31%, −20; 95% CI, −33 to −7. Follow-up (n = 18) reported mean 49-point reduction in stress (95% CI, −72 to −24, *p* = 0.002). *Qualitative comments:* “81% reported that they felt more capable of dealing with their hospitalization, 81% felt more at peace, 71% felt more connected with what is sacred, 96% would recommend chaplain-led picture-guided spiritual care to others, and 0% felt worse after receiving spiritual care.”	Chaplain-led picture-guided spiritual care is feasible among mechanically ventilated adults and shows potential for reducing anxiety during and stress after an ICU admission.
Elham et al. (2015, Iran) [44]	Quasi-experimental	“To investigate the effects of a need-based spiritual/religious intervention on spiritual well-being (SWB) and anxiety of the elderly admitted to the coronary care unit (CCU) of Imam Reza Hospital in Lar, southern Iran.”	*# of participants:* 66 patients in total, with 33 patients each in the intervention group and control group *Mean age:* 68.86 (±8.3) years *Gender:* 39 male, 27 females *Race:* Not reported	**Agent**: Researcher **Recipient**: Elderly patients admitted to the CCU **Content**: Spiritual- and religious-based intervention that included: Caring presence (30 min) Providing hope and encouraging generosity Strengthening personal relationships Providing opportunities for worship and prayer assisting them in performing religious obligations such as ablution, and hanging pictures of natural and relaxing landscapes on the walls of the ward Providing Holy Quran, prayer books Supplying Walkman audio players with relaxing music, prayers, and Quran verses Facilitating patient visits with family and friends Providing small booklets (prepared by researcher) with messages of hope, generosity, and forgiveness from religious scholars **Dose/Intensity**: Length: 60–90 min Frequency: Every evening Duration: At least 3 consecutive days **Delivery Mode**: Face-to-face interaction and provision of religious/spiritual materials **Setting**: CCU **Training**: Not reported.	*Outcome variables:* SWB, anxiety *Measurement tools:* Spielberger State-Trait Anxiety Inventory (S-Anxiety, T-Anxiety), Spiritual Well-Being Scale (SWBS)	Independent and paired *t*-tests, Pearson correlation coefficient, chi-square test, and repeated-measures analysis of variance	*Post-test (Intervention):* SWB scores = 79.03 (±10.95) vs. 83.33 (±9.43) (*p* = 0.001) S-Anxiety scores = 58 vs. 49 (*p* < 0.001). *Post-test (control):* SWB scores = 80.06 (± 10.36) vs. 81.03 (±8.72) S-Anxiety scores = 59 vs. 58.5 *Statistics:* No significant difference was found between the 2 groups (intervention vs. control) before and after the intervention for SWB scores (*p* = 0.049). Significant difference between the intervention and control groups for S-anxiety and T-anxiety (*p <* 0.001).	Individuals with strong religious beliefs and/or spiritual coping strategies have high SWB. This study’s findings demonstrate a negative correlation between SWB and anxiety and that a spiritual/religious intervention may work to increase SWB in older CCU patients.
Heilmann et al. (2016, Germany) [46]	*RCT*	“To evaluate an intervention with individualized information and emotional support before coronary artery bypass grafting (CABG) in a controlled randomized trial.”	*# of participants:* 253 CABG patients in total, with 139 in the intervention group and 114 in the control group *Mean age:* 68 (±10) years *Gender:* 208 (82.2%) male, 45 (17.8%) female *Race:* 238 (94.1%) participants of German nationality, 15 (5.9%) participants of other unspecified nationalities	**Agent**: Trained nurse. **Recipient**: Patients scheduled for CABG **Content**: Psychotherapeutic intervention including: Open dialogue with patient Short relaxation exercise Acceptance of denied negative emotions Reinforcement of confidence in the medical team and positive surgical outcome Differentiation between vigilance vs. cognitive avoidance coping styles Information on postoperative care, pain management, and ICU stay Promoting emotional expression **Dose/Intensity**: Length: 30 min Frequency: One session Duration: Delivered one day prior to CABG **Delivery Mode**: Face-to-face, nurse-led psychotherapeutic session. **Setting**: Coronary Care Unit **Training**: Delivered by a trained nurse based on a specifically developed manual	*Outcome variable:* Anxiety *Measurement tools:* State-Trait Operation Anxiety (STOA-S, STOA-T), Visual Analog Scale (VAS) *Evaluation timeline:* T0—after giving informed consent and before randomization, T1—in the evening after the intervention, and T2—on postoperative day 5	Multivariate analysis of variance, ANOVAs for continuous variables, chi-square tests for categorical variables	*Intervention group:* *T0*—Affective anxiety: M = 10.19 (±3.90). Cognitive anxiety: M = 10.47 (±3.57). VAS: M = 3.53 (±2.88). Trait anxiety: M = 33.03 (±8.35). *T1*—Affective anxiety: M = 9.10 (±3.34). Cognitive anxiety: M = 9.09 (±3.19). VAS: M = 2.86 (±2.50). Trait anxiety: Not reported. *T2*—Affective anxiety: M = 7.97 (±2.68). Cognitive anxiety: M = 8.69 (±2.82). VAS: M = 1.01 (±1.65). Trait anxiety: M = 33.03 (± 8.35) *Control group:* *T0*—Affective anxiety: M = 10.40 (±3.48). Cognitive anxiety: M = 11.26 (±3.53). VAS: M = 3.62 (±2.81). Trait anxiety: M = 35.19 (±9.31). *T1*—Affective anxiety: M = 10.08 (±3.43). Cognitive anxiety: M = 10.38 (±3.47). VAS: M = 3.39 (±2.69). Trait anxiety: Not reported. *T2*—Affective anxiety: M = 8.35 (±3.34). Cognitive anxiety: M = 9.29 (±0.00). VAS: M = 1.51 (±21.88). Trait anxiety: Not reported. *Statistics:* Decreases in affective (F [1,299] = 14.284, *p* < 0.001) and cognitive anxiety (F [1,299] = 17.457, *p* < 0.001) in the intervention group were statistically significant (*p* < 0.001), as were reductions in intervention VAS scores (F [1,299] = 12.207, *p* < 0.001).	This particular intervention is effective at reducing pre- and postoperative anxiety in CABG patients, as opposed to providing them with routine information alone.
Karnatovskaia et al. (2021, United States) [41]	*Quasi-experimental*	“To train and implement a PSBPS program for the critically ill using doulas, and measure acceptance of this intervention through stakeholder feedback.”	*# of participants:* 43 critically ill patients in the intervention group. No control group was identified in this study. *Mean age:* Median age of participants 67 (58–74) years *Gender:* 58% male *Race:* Not reported	**Agent**: Two trained ICU doulas with a combined 15 years of experience. **Recipient**: ICU patients **Content**: PSBPS (Psychological Support and Behavioral Patient Support) intervention that included a training program for doulas. Specific approaches included: Detailed ICU orientation Unit protocols, safety, infection control, and hygiene via online modules Instruction on using an online medical reference Classroom instruction on suggestion training via recorded presentations Role-playing PSBPS scenarios Application of PSBPS to patients in ICU Skills evaluation by reviewer pre- and post-training Qualitative stakeholder feedback collected by a neutral third party **Dose/Intensity**: Length: median 20 min Frequency: Once daily Duration: Median 4 days **Delivery Mode**: Face-to-face intervention delivered by ICU doulas **Setting**: ICU **Training**: Doulas underwent structured PSBPS training, including online modules (20–30 min each), classroom sessions, and role-play (~20 min), with skills evaluated before application.	*Outcome variables:* Doulas training effectiveness *Measurement tools:* Doulas PSBPS evaluation grading rubric divided into ‘poor’, ‘good’, and ‘excellent’, qualitative questionnaires (open-ended questions) for stakeholder feedback	Kirkpatrick model	*Doulas Training* *Pre-training performance:* Doulas performance was mostly in the ‘good’ range following the first standardized evaluation. *Post-training performance:* Doulas performance was mostly ‘excellent’ after training sessions. *Patient feedback:* “93% (n = 40) recalled doulas explaining what was happening to them, and 86% (n = 37) found this comforting. 67% (n = 29) remembered their hand being held; of those, 28 reported it as comforting. When asked what made them feel better while in the ICU, 79% (n = 34) recalled specific aspects with themes and representative responses.” *Family feedback:* Comments from families included those about how the intervention was soothing, helpful, reassuring, and comforting. *Nurse feedback:* Thirty-two bedside nurses commented. Thirty-four percent found that positivity may not reflect reality, it may overstep nurses’ roles, intervention should be longer, and better coordination may be needed. Eighty-one percent found ICU doulas to be helpful. Major themes included the intervention being soothing/relaxing to the patient.	Two doulas were successfully trained and were able to effectively provide PSBPS, as evidenced by the doulas’ graded progress and stakeholder feedback themes. The training was beneficial to the doulas themselves and was also reported to be helpful by patients and families.
Szilágyi et al., 2014 [45]	*RCT*	“To investigate the effect of positive suggestions to ventilated patients in ICU via pre-recorded standard text delivered via the earphones and a simple MP3 player.”	*# of participants*: 26; Control: 6, Music: 6, Suggestion: 6, Rejector: 8 Mean age: 65.34 (16.69) years Gender: 26.92% (% Female *Race:* Not reported	**Agent**: Pre-recorded MP3 messages delivered via headphones (male voice). **Recipient**: Ventilated ICU patients **Content**: Positive suggestion intervention involving structured, reassuring messages promoting recovery and healing. Examples of the recorded suggestions include: “As you begin to notice the signs of your recovery—the movement of tubes, machines, and equipment all centered around you—you come to realize that everything happening is for you, designed to support your healing even more effectively.” “You often experience a pleasant sensation. The way people are working around you, working for you, brings comfort. It’s reassuring to know how important you are to this team.” “You begin to understand that asking something of your body is completely natural. Every cell is working for you—not just out of duty, but because it’s their purpose. Everything is unfolding for your benefit.” **Dose/Intensity**: Length: 30 min Frequency: Once daily Duration: Throughout ICU stay **Delivery Mode**: MP3 and headphones; self-administered under supervision. **Setting**: ICU **Training**: Not applicable (pre-recorded intervention).	*Outcome variables:* Length of mechanical ventilation Length of ICU stay *Measurement tools: Not reported;* presumably recorded from patient charts or the hospital’s electronic medical records system *Timing of assessment:* before and after the intervention	Independent samples *t*-test and analysis of variance	*Mean and SD Length of mechanical ventilation* Unified Control = 232.02 (165.60) Music = 135.33 (96.09) Suggestion = 85.25 (34.92) *Mean and SD Length of stay in ICU* Unified Control = 314.25 (178.40) Music = 187.75 (108.19) Suggestion = 134.24 (73.33) *Statistics:* Huge effect size for length of mechanical ventilation in the positive suggestion group (*d* = 1.46, *p* < 0.06) compared to the control group Very large effect sizes for length of ICU stay in the positive suggestion group as compared to the control group (*d* = 1.26, *p* < 0.07) Post-hoc analysis results showed that the length of ICU stays (134.2 ± 73.3 vs. 314.2 ± 178.4 h) and the time spent on ventilator (85.2 ± 34.9 vs. 232.0 ± 165.6 h) were significantly shorter in the positive suggestion group compared to the unified control.	Positive suggestions, delivered through a simple and accessible method like MP3 players, can be a valuable tool in improving patient outcomes in the ICU setting.
Tan et al. (2020, United States) [42]	*Quasi-experimental*	“To investigate the feasibility of intensivists delivering psychological support based on positive suggestions (PSBPS) to reduce intubated patients’ psychological distress.”	*# of participants:* 76 patients in total. Thirteen in the intervention group and 63 in the control group. *Mean age:* 67 (57–72) years for the intervention group. 61 (53–70) years for the control group. *Gender:* 6 (46%) female in the intervention group. 29 (46%) female in the control group *Race:* Not reported	**Agent**: Three study intensivists. **Recipient**: Intubated patients with expected ICU stays of greater than 48 h **Content**: PSBPS (Psychological Support and Behavioral Patient Support) intervention including: “(1) Physicians developed rapport with patients, (2) Once the patient is off sedation and able to communicate, the team gathers bidirectional connection by having the patient follow simple directions and be more active in treatment, (3) A final debrief- the patient is informed of what to expect after transferring to a recovery ward, fears are explored, and the ICU experience is normalized. Therapeutic touch is also utilized.” **Dose/Intensity**: Length: Median 7 min (range 5–12 min) Frequency: Once daily Duration: entire ICU stay; median 4 days (IQR 2–6) **Delivery Mode**: Face-to-face, intensivist-led sessions at the patient’s bedside. **Setting**: ICU **Training**: Not reported	*Outcome variables:* Feasibility (primary outcome), anxiety, depression, stress *Measurement tools:* Hospital Anxiety and Depression Scale (HADS-A, HADS-D), Montreal Cognitive Assessment-Blind (MoCA-BLIND), Impact of Events Scale-Revised (IES-R)	Wilcoxon signed-rank test, Pearson chi-square test, Fisher’s exact test, linear regression models	*Post-test (Intervention):* MoCA-BLIND score = 16 (13 to 20). HADS-D score = 5 (4 to 9). HADS-A score = 6 (3 to 9). IES-R intrusion score = 0.9 (0.1 to 1.9). IES-R avoidance score = 1.1 (0.1 to 2.3). IES-R hyperarousal score = 1.3 (0.2 to 1.7). *Post-test (Control):* MoCA-BLIND score = 17 (15 to 19). HADS-D score = 6 (3 to 9). HADS-A score = 7 (5 to 11). IES-R intrusion score = 1.0 (0.5 to 1.9). IES-R avoidance score = 0.8 (0.3 to 1.4). IES-R hyperarousal score = 1.0 (0.5 to 2.0). *Statistics:* MoCA-BLIND < 18 odds ratio 1.5 (*p* = 0.95). HADS-D ≥ 8 odds ratio 0.74 (*p* = 0.64). HADS-A ≥ 8 odds ratio 0.38 (*p* = 0.24). IES-R < 1.6 odds ratio 1.04 (*p* = 0.95).	This intervention was feasible, as evidenced by the high completion rate, minimal session interruptions, absence of adverse events, and acceptance from patients and their family members. Positive suggestion interventions coupled with medical treatment may be an effective method of reducing psychological distress in mechanically intubated patients. A larger randomized study may be needed to confirm the efficacy of PSBPS.
Ong et al. (2020, United States) [43]	*Quasi-experimental*	“To evaluate the feasibility and efficacy of using meditative virtual reality (VR) to improve the hospital experience of ICU patients.”	*# of participants:* 46 patients in the intervention group. No control group was identified in this study. *Mean age:* 50 (±18) years *Gender:* 30 (65%) male *Race:* 8 (18%) Black, 36 (78%) White, 2 (4%) other unspecified ethnicities	**Agent**: Study staff. **Recipient**: ICU patients from surgical and trauma ICUs **Content**: Guided virtual VR-based meditative intervention that “consisted of a smartphone placed in a Google Daydream (vr.google.com/daydream) headset and a pair of Bluetooth headphones”, collectively referred to as Digital Rehabilitation Environment Augmenting Medical System (DREAMS). ‘Google Spotlight Stories’ Pearl” (atap.google.com/spotlight-stories) was used to provide an orientation to VR, and ‘RelaxVR’ was also used to “provide patients with a calm immersive scene with voice-guided meditation that promoted breath control and relaxation”. **Dose/Intensity**: Length: 5–20 min Frequency: Once daily Duration: 7 days **Delivery Mode**: VR headset and audio-guided meditation; staff-assisted. **Setting**: Surgical and trauma ICU **Training**: Not reported	*Outcome variables:* Pain, sleep quality, affect, and delirium state *Measurement tools:* Defense and Veterans Pain Rating Scale (DVPRS), Richards-Campbell Sleep Questionnaire (RCSQ), Hospital Anxiety and Depression Scale (HADS0, Confusion Assessment Method for the ICU (CAMS-ICU). Questionnaire also administered to collect qualitative comments.	Paired *t*-tests and mixed models	*Pain:* Dosage and frequency of opioid medications decreased at a rate of 12.9%. (*p* > 0.05, 95% CI, 21.7–4.03) *Sleep:* RCSQ score improved by 4.56 (*p* > 0.05, 95% CI, 1.06–8.06). *Affect:* statistically significant decreases in anxiety (estimate = 2.17; *p* < 0.05, 95% CI, –4.23 to –0.106) and depression (estimate = −1.25, *p* > 0.05, −95% CI, –2.37 to –0.129) were noted from before the first exposure to before the third exposure *Delirium:* 13 patients of a total of 46 were delirious at least 1 day during admission. Seven participants were delirious prior to study but recovered before enrollment and remained non-delirious. Six became delirious after DREAMS. *Qualitative comments:* Participants found DREAMS to be comfortable, enjoyable, and helpful for pain management. They expressed mixed feelings as to whether it helped them sleep better or not. Common themes included novelty of VR, emotionally evocative experiences, relaxation, technical frustrations, and temporary effects.	The use of meditative VR technology in the ICU was generally well-received by patients. The intervention improved patients’ experiences in the ICU by reducing anxiety and depression.
Wade et al. (2018, United Kingdom) [48]	*Mixed Methods Study*	To develop and test the feasibility of a psychological intervention to reduce acute stress and prevent future morbidity in critical care patients.	*# of participants:* Intervention feasibility study—127 patients. Trial feasibility study—86 patients. No control group was identified in this study. *Mean age:* Not reported *Gender:* Not reported *Race:* Not reported	**Agent**: Trained POPPI (Provision Of Psychological support to People in Intensive care) nurses. **Recipient**: ICU patients **Content**: Psychological support program including: Creation of a therapeutic environment Identification of patients with acute stress Delivery of three stress support sessions Relaxation and recovery program Dose/Intensity: Median session lengths: 35 min (Session 1), 30 min (Sessions 2 and 3) Frequency: Ideally three sessions; 39 patients received 2–3 sessions, 5 received 1 session Duration: Sessions began within 48 h of IPAT screening; three sessions were ideally completed within one week **Delivery Mode**: Face-to-face, nurse-led sessions at patient bedside. **Setting**: ICU. **Training**: POPPI nurses are trained via course materials to deliver intervention and create a therapeutic environment.	*Outcome variables:* Feasibility and acceptability, stress *Measurement tools:* Nurse and patient feasibility questionnaires, focus groups administered to nurses, “stress thermometers” on which patient’s reported their stress levels from 0 to 10 at the beginning and end of each stress support session, PTSD Symptom Severity Scale-Self-Report (PSS-SR), and the 10-Item Centre for Epidemiologic Studies Depression Scale (CES-D)	Patient satisfaction questionnaires	*Intervention feasibility study:* All 10 POPPI nurses rated the face-to-face course as ‘good’ with nine (90%) rating relevance to their new role as ‘good’. 14 (93%) patients rated their satisfaction with stress support sessions as ‘good’. Twelve (80%) rated the number and duration of sessions as ‘just right’. Ten (67%) patients found they learned good coping strategies from the program. Stress reduced by a median of 3 points (IQR 1–5 points) from the start of the first session to the end of the third session among the 25 patients who received all three sessions. *Trial feasibility study:* “Completeness of the primary outcome measure (PSS-SR) was very good. From 1054 fields, only 24 (2.3%) had missing data.” *Qualitative comments:* Nurses found that the stress support sessions were welcomed and rewarding for patients and staff. They reported that the training had raised awareness, changed staff thinking, and led to better unit environments. Patient feedback on the stress support sessions included “every hospital should have it”, “it seemed a life saver”, and “I hope POPPI will eventually be used in all ICUs”.	The POPPI psychological intervention to reduce stress and prevent long-term morbidity for critical care patients was feasible, acceptable, and ready for evaluation in a cRCT.
Yang et al. (2020, China) [47]	*Quasi-experimental*	“To explore the relationship between psychosocial support-related factors and the mental health of COVID-19-positive patients.”	*# of participants:* 35 patients in the intervention group. No control group was identified in this study. *Mean age:* 57.00 (±13.44) years *Gender:* 21 (60%) male, 14 (40%) female *Race:* Not reported	**Agent**: Trained psychotherapist and nurse. **Recipient**: COVID-19-positive patients admitted to an isolated ICU **Content**: Psychological, face-to-face intervention focusing on relaxation and positive support. Specific approaches included: Evaluation of psychosocial impact of disease on patients and their family members and close friends Listening and providing positive attention Supportive psychotherapy and empathy Muscle and breath relaxation Cognitive-behavioural therapy (CBT) **Dose/Intensity**: Length: 15–30 min per session Frequency: 3 times per week Duration: Not reported **Delivery Mode**: In-person consultation **Setting**: Isolated ICU **Training**: Delivered by a trained psychotherapist and nurse; specific training not reported.	*Outcome variables:* Sleep, depression, anxiety, and social support *Measurement tools:* Chinese version of the Pittsburgh Sleep Quality Index (PSQI), Patient Health Questionnaire (PHQ-9), Generalized Anxiety Disorder Assessment (GAD-7), Social Support Rate Scale (SSRS)	ANOVA test, chi-square test, and linear regression models	*Pre-test* vs. *post-test:* Mean PSQI: 11.20 (±5.06) vs. 49.16 (±31.27), *p* < 0.0001 Mean PHQ-9 = 8.80 (±4.26) vs. 52.15 (±42.35), *p* < 0.0001 Mean GAD-7 score = 10.69 (±5.62) vs. 63.86 (±42.62), *p* < 0.0001). Mean SSRS score = 25.57 (±4.60) vs. 29.94 (±3.15), *p* = 0.046 *Statistics:* Improved sleep quality was noted after intervention and was positively associated with improvement from COVID-19 (*p* = 0.000) and better social support (*p* = 0.046).	“Physical and psychological well-being and sleep are affected by many social support-related factors”. Administering psychological interventions to COVID-19 patients in countries outside of China may also prove beneficial.

Note. Data are extracted directly from the referenced publications without modification; no author interpretation has been applied.

**Table 2:** Appraisal of identified studies using the National Institute for Health and Clinical Excellence (NICE) Checklist.

Author and Date	Bannon et al. (2020) [39]	Berning et al. (2016) [40]	Elham et al. (2015) [44]	Heilmann et al. (2016) [46]	Karnatovskaia et al. (2021) [41]	Ong et al. (2020) [43]	Szilágyi et al. (2014) [45]	Tan et al. (2020) [42]	Wade et al. (2018) [48]	Yang et al. (2020) [47]
*Is the source population or source area well described?*	+	+	+	++	−	++	++	++	++	++
*Is the eligible population or area representative of the source population or area?*	++	++	++	++	−	++	++	++	++	+
*Do the selected participants or areas represent the eligible population or area?*	++	++	++	++	−	+	++	++	++	+
*Was selection bias minimised?*	++	NR	NR	++	NR	NR	++	−	+	NR
*Were interventions (and comparisons) well described and appropriate?*	++	++	+	++	++	+	++	++	+	+
*Was the allocation concealed?*	++	NR	NR	++	NA	NA	++	−	NR	NR
*Were participants or investigators blind to exposure and comparison?*	−	NA	NR	+	NA	NA	++	−	NR	NA
*Was the exposure to the intervention and comparison adequate?*	+	NA	++	+	NA	NA	++	+	+	NA
*Was contamination acceptably low?*	++	NA	++	++	NA	NA	++	++	NR	NA
*Were other interventions similar in both groups?*	++	NA	++	++	NA	NA	++	++	NA	NA
*Were all participants accounted for at study conclusion?*	−	++	−	−	++	NR	++	++	++	NR
*Did the setting reflect standard Canadian practice?*	++	++	++	++	++	++	++	++	++	+
*Did the intervention or control comparison reflect standard Canadian practice?*	++	++	++	+	+	++	++	++	++	++
*Were outcome measures reliable?*	+	+	+	+	+	+	++	+	+	+
*Were all outcome measurements complete?*	++	++	+	++	−	++	+	++	+	++
*Were all important outcomes assessed?*	+	+	+	+	+	+	+	+	+	+
*Were outcomes relevant?*	++	++	++	++	++	++	++	++	++	++
*Were there similar follow-up times in exposure and comparison groups?*	++	NA	++	++	NA	NA	NA	++	NA	NA
*Was follow-up time meaningful?*	+	++	+	+	++	++	NA	++	NR	+
*Were exposure and comparison groups similar at baseline? If not, were these adjusted?*	++	NA	++	++	NA	NA	++	++	++	NA
*Was intention to treat (ITT) analysis conducted?*	NR	NR	NR	NR	NA	NR	NR	NR	NR	NR
*Was the study sufficiently powered to detect an intervention effect (if one exists)?*	NR	NR	−	++	NR	NR	−	−	NR	NR
*Were the estimates of effect size given or calculable?*	NR	NR	NR	+	NR	NR	++	NR	NR	NR
*Were the analytical methods appropriate?*	++	+	+	++	+	+	++	+	−	+
*Was the precision of intervention effects given or calculable? Were they meaningful?*	++	+	+	++	NA	+	++	++	−	+
*Are the study results internally valid* (i.e., *unbiased*)?	+	+	−	++	+	+	++	+	+	+
*Are the findings generalizable to the source population* (i.e., *externally valid*)?	+	++	+	++	−	+	++	+	+	+

++ the study has been designed or conducted in such a way as to minimize the risk of bias. + either the answer to the checklist question is not clear from the way the study is reported, or the study may not have addressed all potential sources of bias for that particular aspect of study design. − significant sources of bias may persist. NR: Not reported—study under review fails to report how they have (or might have) been considered. NA: Not applicable—study design aspects that are not applicable given the study design under review.

**Table 3:** Summary of Interventions, Outcomes, and Study Quality.

Author (Year, Country)	Study Design	Intervention Type	Outcome	Study Quality
Bannon et al. (2020, United States) [39]	Randomized Controlled Trial (RCT)	Relaxation-based intervention with family caregiver	Decreased anxiety and post-traumatic stress in both stroke patients and caregivers during ICU	Moderate
Berning et al. (2016, United States) [40]	Quasi-experimental	Spiritual-religious-based intervention	Reduced anxiety and stress in mechanically ventilated patients	Low
Elham et al. (2015, Iran) [44]	Quasi-experimental	Spiritual- and religious-based intervention	Increased spiritual well-being and decreased anxiety in older coronary care unit patients	Low
Heilmann et al. (2016, Germany) [46]	RCT	Psychological support by trained nurses	Reduced pre- and postoperative anxiety in Coronary Artery Bypass Grafting (CABG) patients	Moderate
Karnatovskaia et al. (2021, United States) [41]	Quasi-experimental	Positive suggestions	After training, doulas can effectively provide reassuring support, thereby reducing ICU patients’ distress.	Moderate
Szilágyi et al., 2014 [45]	RCT	Positive suggestions via prerecorded messages	Reduced length of ICU stays and the time spent on ventilator	High
Tan et al. (2020, United States) [42]	Quasi-experimental	Positive suggestions	Reduced psychological distress in mechanically intubated patients	Low
Ong et al. (2020, United States) [43]	Quasi-experimental	Guided virtual VR-based relaxation intervention	Reduced anxiety and depression in surgical and trauma ICUs	Low
Wade et al. (2018, United Kingdom) [48]	Mixed Methods Study	Psychological support by trained staff	Improved coping and reduced stress	Moderate
Yang et al. (2020, China) [47]	Quasi-experimental	Psychological, face-to-face intervention focusing on relaxation and positive support	Improved sleep quality, depression, and anxiety in isolated ICU patients with COVID-19	Low

**Table 4:** Measures of effect of identified interventions.

Author, Year	Intervention	Psychological Outcome	EFFECT SIZE
**A. Relaxation-based Interventions**			
Bannon et al., 2020 [39]	Recovering Together	Anxiety PTS	*d* = −1.25 *d* = −0.83
Ong et al. (2020) [43]	virtual reality-based meditative intervention	Anxiety Depression	inadequate data to calculate effect size
**B. Psychotherapy-based Interventions**			
Yang et al. (2020) [47]	in-person relaxation and support	Depression Anxiety Social support	inadequate data to calculate effect size
Wade et al. (2018) [48]	Psychological support	Stress Coping	inadequate data to calculate effect size
Heilmann et al. (2016) [46]	psychotherapy to provide emotional support	*Immediately After intervention*:	
		cognitive anxiety	*d* = −0.39
		affective anxiety	*d* = −0.125
		VAS	*d* = −0.20
		*On postoperative day 5*:	
		VAS	*d* = −0.28
		affective anxiety	*d* = −0.125
		Cognitive anxiety	*d* = −0.17
**C. Spirituality-based interventions**			
Elham et al. (2015) [44]	spiritual- and religious-based interventions	SWB S-anxiety T-anxiety	inadequate data to calculate effect size
Berning et al. (2016) [40]	spiritually based interventions	Anxiety Stress	inadequate data to calculate effect size
**D. Positive suggestion-based interventions**			
Tan et al. (2020) [42]	psychological support based on positive suggestions	Anxiety Depression Stress	inadequate data to calculate effect size
Karnatovskaia et al., 2021 [41]	Psychological Support Based on Positive Suggestions	Stress Coping	inadequate data to calculate effect size

## Data Availability

The data supporting the findings of this study are available within the article.

## Appendix A

### Appendix A.1. Search Strategy (OVID/MEDLINE)

**Table:**

SN	Searches	Results
1	((“Intensive care” or “critical care”) adj3 (unit* or ward* or floor* or nurs*)).mp.	530,360
2	“critical* ill*”.mp.	202,608
3	ICU.ti,ab.	241,335
4	1 or 2 or 3	718,543
5	patient*.mp.	21,576,739
6	(((Psychological* or mental* or emotion*) adj3 (stress or distress or pressure or strain or tension)) or anxiet* or anxiousness or worry or worrie* or nervous*).mp.	3,215,392
7	(((Psychosocial or spiritual* or religious or religion) adj3 (intervention* or support*)) or counsel* or “positive suggestion*” or “positive messag*” or mindfulness or meditat*).mp.	687,587
8	4 and 5 and 6 and 7	626
9	(neonat* or p?ediatric* or child* or infant*).mp.	8,285,783
10	8 not 9	444
11	remove duplicates from 10	334
12	11 and 2009:2023.(sa_year).	232
